# UHPLC-MS Phytochemical Profiling and Insight into Bioactivity of *Rabelera holostea* (Greater Stitchwort) Extract

**DOI:** 10.3390/molecules28031274

**Published:** 2023-01-28

**Authors:** Jelena S. Katanić Stanković, Jelena Đorović Jovanović, Danijela Mišić, Uroš Gašić, Stefanie Nikles, Zoran Marković, Rudolf Bauer

**Affiliations:** 1 Department of Science, Institute for Information Technologies Kragujevac, University of Kragujevac, Jovana Cvijića bb, 34000 Kragujevac, Serbia; 2 Department of Plant Physiology, Institute for Biological Research “Siniša Stanković”—National Institute of Republic of Serbia, University of Belgrade, Bulevar Despota Stefana 142, 11060 Belgrade, Serbia; 3 Department of Pharmacognosy, Institute for Pharmaceutical Sciences, University of Graz, Beethovenstrasse 8, 8010 Graz, Austria

**Keywords:** *Rabelera holostea* (L.) M. T. Sharples & E. A. Tripp, *Stellaria holostea*, greater stitchwort, UHPLC, phenolics, bioactivity, anti-inflammatory activity, molecular docking

## Abstract

*Rabelera holostea* (L.) M. T. Sharples & E. A. Tripp (Greater Stitchwort), formerly known as *Stellaria holostea* L., is widespread in the warm temperate areas of Europe and Western Asia, the Caucasus region, as well as in some countries of North Africa. Nowadays it is considered as a weed, but earlier it was often used raw in salads or for the treatment of various inflammatory disorders. The goal of this study was to determine the constituents of the methanol extract of *R. holostea* aerial parts and its biological potential in terms of antioxidant, antimicrobial, and anti-inflammatory properties. Until now, the constituents and biological activities of this plant were not reported in detail. A comprehensive phytochemical profiling of the extract has shown that phenolic acids, such as ferulic, chlorogenic, and *p*-coumaric acid, flavonoids and flavonoid glucosides, such as chrysoeriol, rutin, and naringin, are the most abundant compounds. The antioxidant activity of *R. holostea* extract towards DPPH and ABTS radicals, but also the total antioxidant capacity and the inhibition of lipid peroxidation were moderate. The antimicrobial potential was pronounced mostly towards some fungi such as *F. oxysporum* (MIC 1.25 mg/mL), whereas the capacity of *R. holostea* to affect the growth of bacteria was much less pronounced. *R. holostea* extract was most inclined to anti-inflammatory activity. At a concentration of 50 µg/mL, it significantly inhibited both cyclooxygenase enzymes (COX-1 and COX-2) by 71.24% and 72.83%, respectively. Molecular docking studies indicated that chlorogenic acid and chrysoeriol are the main contributors to COX-1 and COX-2 inhibitory activity.

## 1. Introduction

**Figure 1:**
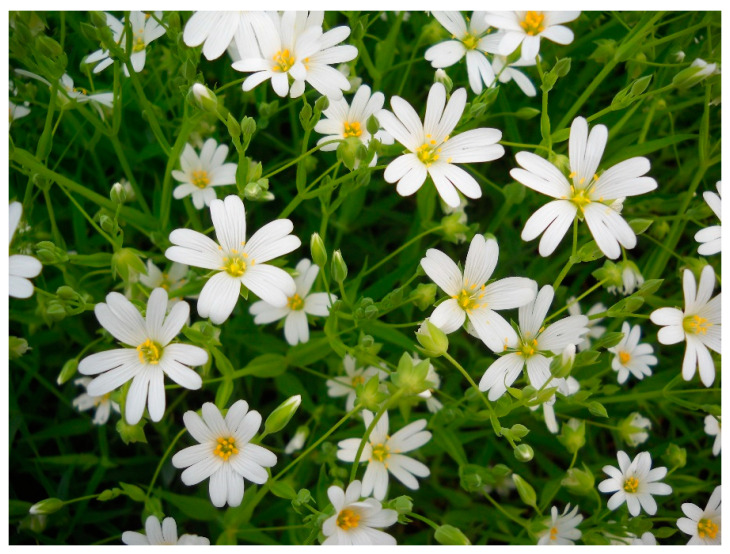
*Rabelera holostea* (L.) M. T. Sharples & E. A. Tripp (Caryophyllaceae) aerial parts with flowers (Photo by: J. S. Katanić Stanković, April 2015).

*Rabelera holostea* (L.) M. T. Sharples & E. A. Tripp (greater stitchwort, Figure 1), formerly known as *Stellaria holostea* L., belongs to the family Caryophyllaceae, whose members are generally characterized by the presence of C-glycosyl-flavonoids with numerous biological activities [1]. According to Royal Botanical Gardens (KEW), the native range of this species is Europe up to West Siberia, the Middle East, and Northwest Africa. It is a perennial and rhizomatous geophyte and grows primarily in temperate biomes [2]. *R. holostea* grows in bright meadows and on the edges of forests, along roads, and in neglected and shady places. It can be found from the lowlands to 1800 m above sea level. The name of the genus to which it previously belonged, *Stellaria*, comes from the Latin word “*stella”* (star) because of the star-shaped flower petals. The species name *holostea* is derived from the Greek word “*holosteon”* meaning whole bone. The aerial parts of the plant are edible and young shoots and leaves are especially tasty, thus they have been used raw as a salad or prepared by cooking in the same way as spinach [3].

To understand the properties and application of the investigated plant species *Rabelera holostea*, first, we must refer to the genus *Stellaria* (Caryophyllaceae), to which, until recently, this plant belonged. Until 2019, it was classified under the name *Stellaria holostea* L. More than 100 plant species belong to this genus but most of them have not been sufficiently investigated in terms of phytochemical composition and potential biological activities. The most widely studied *Stellaria* species is *Stellaria media* (L.) Vill. (common chickweed), whose biological potential and chemical composition has been investigated in more detail. *S. media* is an annual herbaceous weed that grows widely in cold and temperate regions of Europe, Asia, and Northern America [4,5]. It is quite rich in vitamins, minerals, polyphenols, triterpenoids, and beta-carotene [6]. Since the nutritive properties of *S. media* are superb, it is used fresh as a salad [4]. It has a long tradition of ethnopharmacological use in the Eurasian region, mainly for some skin disorders, to treat cuts, bruises, and burns [7], but also to treat many inflammatory processes, such as digestive, respiratory, and reproductive tracts, bronchitis, asthma, rheumatic pains, arthritis, period pain, diarrhea, measles, and it is also used to reduce swelling [5,7,8]. *R. holostea* (greater stitchwort) is used similarly for the treatment of skin diseases, as an anti-inflammatory agent, but also for its anti-rheumatic, anti-hypertensive, and expectorant properties [1].

In 2019, Sharples and Tripp [9] published a study on phylogenetic relationships within and delimitation of the cosmopolitan flowering plant genus *Stellaria* L. (Caryophyllaceae). They suggested some modifications, which, among others, refer to the “description of a new genus, *Rabelera*, to accommodate the lineage previously and more widely known as *Stellaria holostea*”. This is how symbolically this plant species has become “a fallen star” by replacing the genus *Stellaria* with *Rabelera*.

The goal of this investigation was to properly address all benefits and biological potential (antioxidant, antimicrobial, and anti-inflammatory) of the *R. holostea* aerial part methanol extract using *in vitro* and *in silico* methodologies. Another goal was to characterize the chemical composition of the tested extract in terms of identification and quantification of secondary metabolites using UHPLC-MS^4^ Orbitrap and UHPLC-DAD/±HESI-MS/MS techniques. Since this plant species has been quite neglected but is very widespread, the intention was to show that it could gain new uses based on its health benefits and therapeutic potential.

## 2. Results

### 2.1. Phytochemical Profile of R. holostea

**Figure 2:**
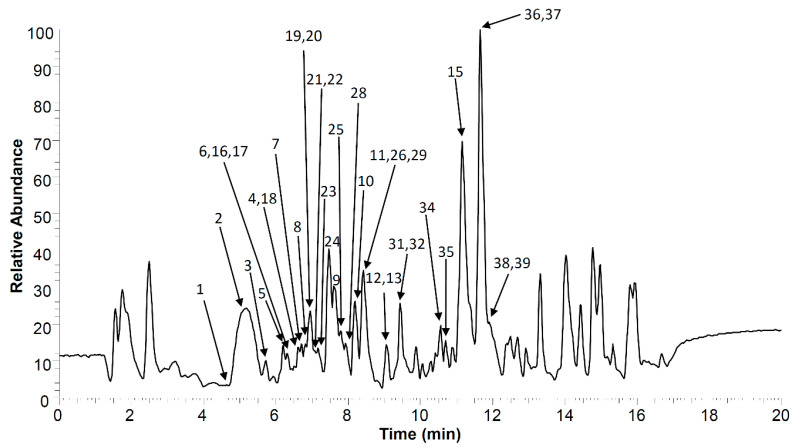
UHPLC-MS^4^ Orbitrap chromatogram (negative ionization mode) of *R. holostea* aerial part methanolic extract. Compounds **14**, **27**, **30** and **33** are not visible in this plot, as their peaks are below the baseline.

The qualitative analysis of *R. holostea* extract phytochemicals was done by UHPLC-high-resolution mass spectrometry (HRMS) in combination with MS^4^ fragmentation. The UHPLC-MS^4^ Orbitrap metabolic chromatogram of *R. holostea* aerial part extract is presented in Figure 2, and all obtained data regarding the peak numbers, retention times (*t*_R_, min), compound names, molecular formulas, calculated and exact masses ([M–H]^−^, *m*/*z*), mean mass accuracy errors (Δ mDa), as well as major MS^2^, MS^3^ and MS^4^ fragment ions of compounds in *R. holostea* extract, are listed in Table 1. There are 39 phenolic compounds that could be identified. These can be roughly divided into 5 categories; (1) 4 hydroxybenzoic acids, (2) 11 hydrocinnamic acids, (3) 12 flavonoid *C*-glycosides, (4) 5 flavonoid *O*-glycosides, and (5) 7 flavonoid aglycones.

Of the four hydroxybenzoic acid derivatives, **1** and **4** were confirmed by reference standards, and **2** and **3** were identified as hexosides of dihydroxybenzoic acids giving specific fragmentation of the loss of the hexosyl group (162 Da).

Within the 11 hydrocinnamic acid derivatives, free acids (**9**, **11**–**13**), methyl (**15**) and quinic acid esters (**5**, **7**, and **8**), 1 glycoside (**6**) and 1 phenylethanoid glycoside—verbascoside (**10**), were detected. Verbascoside is a derivative of caffeic acid that also contains the phenylethanoid hydroxytyrosol, hexose and rhamnose in its structure, and its fragmentation was in accordance with previously published data [10].

**Figure 3:**
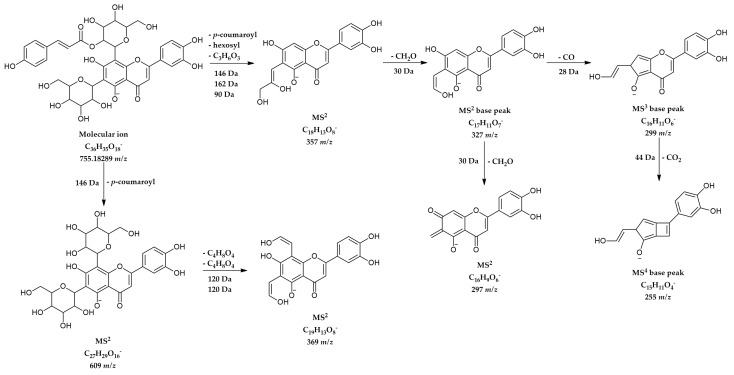
The chemical structure and proposed MS fragmentation pathway of compound **27**.

Identification of all 12 derivatives of flavonoid *C*-glycosides was done through HRMS and MS^n^ data, following specific rules of homolytic cleavage of *C*-bond sugar units [11]. All compounds from this group are flavone derivatives of apigenin and luteolin. Compound **27** at 8.57 min and 755 *m*/*z* was identified as luteolin 6-*C*-hexoside-8-*C*-(2”-coumaroyl)-hexoside, because its fragmentation was consistent with previously published data on the analysis of *C*-glycosyl flavones from *Spergularia rubra* (Caryophyllaceae) [12]. The chemical structure and proposed fragmentation pathway of this compound are shown in Figure 3.

Among flavonoid *O*-glycosides, compounds **28**–**30** were identified using available reference standards; compounds **31** and **32** were confirmed by examination of their MS spectra, and found to be jaceosidin derivatives, specific for *Paronychia argentea* (Caryophyllaceae) [13].

The presence of all identified flavonoid aglycones (compounds **33**–**39**) was confirmed by comparing its MS data with reference standards.

The quantification of 17 targeted phytochemicals in extracts of *R. holostea* aerial parts was done by applying UHPLC(-)HESI-MS/MS analysis. The results are presented in Table 2, with retention times (t_R_), parent and product ions, and content is expressed in milligrams of compound per kilogram of the extract.

Through quantitative analysis, it was concluded that the most abundant compounds in the methanolic extract of *R. holostea* aerial parts were phenolic acids, first of all, *p*-coumaric acid (81.18 mg/kg), followed by chlorogenic (46.35 mg/kg) and ferulic (42.98 mg/kg) acids. The concentrations of these three phenolic acids in the tested extract were much higher than all other compounds that were determined. Regarding the flavonoid content, the highest value was recorded for chrysoeriol (luteolin 3′-methyl ether) 3.83 mg/kg, followed by rutin and naringin (3.41 and 1.16 mg/kg, respectively). All other compounds were present in much lower amount compared to the aforementioned. Since the values of total flavonoid content expressed as rutin equivalents were even higher, also other flavonoids contributed, which were identified in the extract but not quantified, e.g., catechin, naringenin, hyperoside, eriodictyol, apigenin, kaempferol, and also luteolin and apigenin derivatives.

### 2.2. In Vitro Biological Activities of R. holostea

The *in vitro* biological properties of the methanol extract of *R. holostea* aerial parts were evaluated via three different sets of experiments, in order to assess the potential towards the neutralization of free radicals and total antioxidant capacity, present antibacterial and antifungal potential, and demonstrate anti-inflammatory action by inhibiting the activity of selected enzymes included in the inflammatory response.

The antioxidant activity was evaluated using generated free radicals DPPH^·^ and ABTS^·+^, but also using a lipid peroxidation model system and total antioxidant capacity. The results of the applied assays are listed in Table 3. The methanol extract exerted moderate antioxidant effects in all applied assays. The lowest IC_50_ value was observed against DPPH radical (IC_50_ 246.7 μg/mL), followed by ABTS^·+^ scavenging activity, and the potential to inhibit lipid peroxidation chain reactions (IC_50_ 420.4 and 570.4 μg/mL, respectively). The results were significantly (*p* < 0.05) different and much lower than those of respective antioxidants used as standards, caffeic acid, quercetin, and butylated hydroxytoluene (BHT). The results showed that one gram of the methanolic extract of *R. holostea* possesses the antioxidant capacity of 192 mg of ascorbic acid (vitamin C).

As for the antimicrobial potential of the methanol extract of *R. holostea* aerial parts, the antibacterial and antifungal properties were monitored. The antimicrobial action of the extract on eight selected bacteria (4 Gram-positive and 4 Gram-negative species) and the same number of fungal species, was expressed as a minimal inhibitory concentration, compared to the standard antibiotic and antimycotic (chloramphenicol and ketoconazole), respectively, and is presented in Table 4.

The tested extract showed moderate antibacterial activity with MIC values between 5 × 10^3^ and 20 × 10^3^ μg/mL (5–20 mg/mL). The results were not particularly dependent on whether the bacterium was G+ or G−, thus the most sensitive ones were *E. faecalis* and *A. chroococcum*, with the same MIC value of 5 mg/mL. *R. holostea* extract, at the highest applied concentration (20 mg/mL), did not have any influence on the growth and development of *P. aeruginosa*, whereas the same concentration was effective against *B. mycoides*. Regarding the selected fungal species, the extract exerted a similar level of antimicrobial potential. The acceptable antifungal properties *R. holostea* extract showed against *P. fastigiata* and *P. canescens* (MIC 5 mg/mL) and somewhat better against *F. oxysporum* with MIC value 1.25 mg/mL. At quite high concentrations of 10 and 20 mg/mL the extract showed antifungal properties against other tested fungi. The values of referent compounds, chloramphenicol and ketoconazole, were tens times’ lower compared to the MICs of the extract which is consistent with the fact that they are standard substances that have a clearly defined structure, are used in pure form, not as a mixture, and have proven antimicrobial effects with a clearly explained and defined mechanism of action.

**Figure 4:**
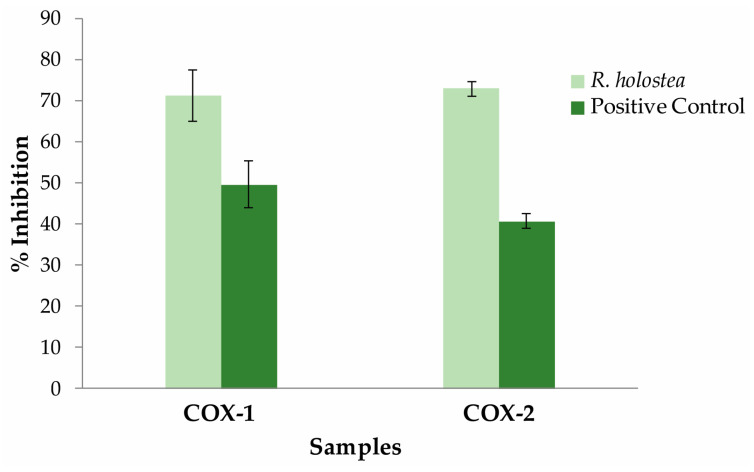
The inhibition of *R. holostea* methanolic extract (50 μg/mL) towards COX-1 and COX-2 activities. The results are from two independent experiments (n = 4, mean ± SD). Positive controls: Indomethacin (1.25 μM) for COX-1 and NS-398 (5 µM) for COX-2, according to Katanić et al. [14].

The anti-inflammatory properties of *R. holostea* aerial part methanol extract were tested *in vitro* through the evaluation of potential activity inhibition of cyclooxygenase-1 and -2 (COX-1 and COX-2). The obtained results were expressed as percentages of inhibition and are presented in Figure 4.

At a concentration of 50 µg/mL, the tested extract showed quite a high inhibitory potential on COX-1 activity with 71.24% of inhibition. In the same assay, the reference compound indomethacin, at a concentration of 1.25 μM, showed much lower inhibition of COX-1 activity (49.63%). A much more significant segment of the effectiveness of the examined extract is its potential to inhibit the activity of COX-2 cyclooxygenase isoform. The results of another *in vitro* assay based on COX-2 activity reported that *R. holostea* extract, used at the same concentration as the previous, manifested high inhibition of COX-2 enzymatic activity with almost the same percentage values (72.83%) compared with the previous one.

As a positive control in COX-2 assay was used NS-398 (5 µM). The results obtained from the reference component (40.72%) indicate that COX-2 inhibition of the tested extract was more than 1.7 fold higher than the used compound NS-398. However, these comparisons should be approached with caution because they are about significantly different concentrations that were applied. It should also be emphasized that the comparison was made between pure compounds, with proven inhibitory activity and defined mechanism of action, and a mixture of different compounds found in the plant extract.

### 2.3. In Silico Examination of Anti-Inflammatory Activity

**Figure 5:**
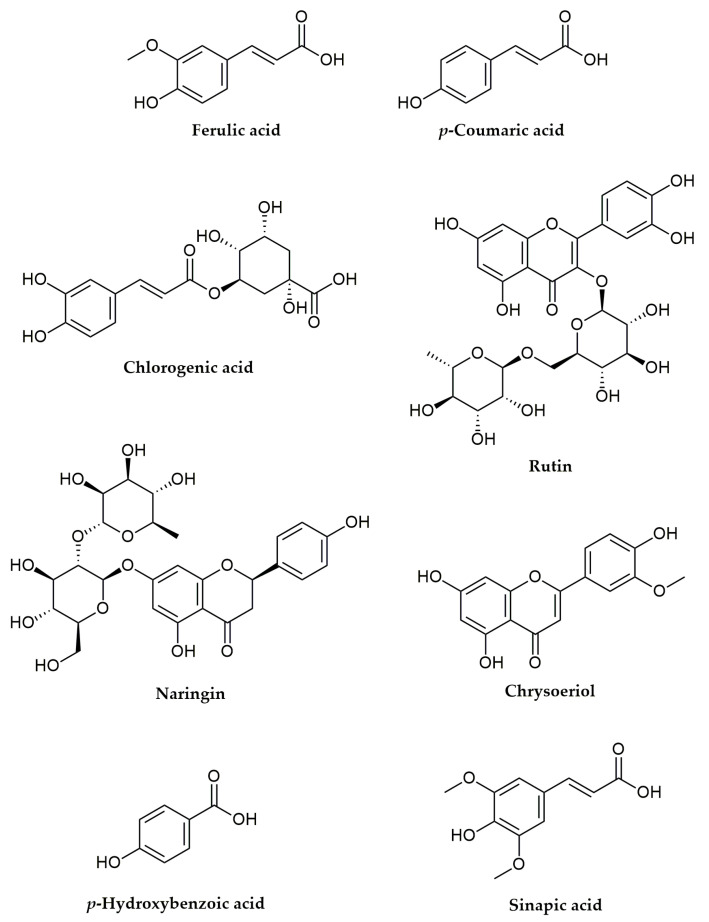
Chemical structures of polyphenolic compounds from *R. holostea* extract analyzed *in silico* for anti-inflammatory activity.

Since the investigated extract showed noteworthy anti-inflammatory activity through *in vitro* studies, the influence on the activity of COX-1 and COX-2 enzymes of the dominant phenolic compounds, which were quantified in the *R. holostea* extract (*p*-coumaric acid (*p*-CA), ferulic acid (FA), chlorogenic acid (CA), *p*-hydroxybenzoic acid (*p*-HBA), sinapic acid (SIN), chrysoeriol (CHR), naringin (NAR), and rutin (RU)) (Figure 5), was evaluated in an *in silico* study.

At the beginning of the study, molecular docking simulations were performed between the indomethacin (IND) and COX-1 enzyme, as well as between NS398 and COX-2 enzyme. The IND and NS398 were included in the present research as model systems since both of them are known inhibitors of COX-1 and COX-2, respectively [15,16].

Clarification of the inhibitory potency of the investigated compounds treated as ligands in molecular docking simulations toward COX-1 and COX-2 was achieved with a careful inspection of the established interactions. Here were considered and discussed the binding modes of protein-ligand complexes that display the best inhibitory potency. The obtained thermodynamic parameters from molecular docking simulations for all tested compounds are listed in Table 5 and Table 6.

The established contacts in the most stable conformations of complex structures of investigated compounds with COX-1 and COX-2 are presented in Supplementary Material (Supplementary Figures S1–S6). The compounds with the lowest values of ∆G_bind_ and K_i_ possessed the highest binding affinity to the targeted proteins and had considerable inhibitory efficacy. Additionally, the lower K_i_ values demonstrated a higher binding affinity and showed that a lesser quantity of a compound was needed to inhibit the receptor’s function. The results from Table 5 and Table 6 indicated the relationship between the values of ∆G_bind_ and K_i_. Specifically, the lower values of ∆G_bind_ were accompanied by lower values of K_i_. The detailed examinations of the obtained results undoubtedly specified CA as the compound with the highest inhibitory potency against COX-1 and COX-2. It was noticed that calculated values ∆G_bind_ and K_i_ for CA were lower than values obtained for referent inhibitors. Namely, the molecular docking simulations of COX-1-CA protein-ligand complex obtained lower values of the ∆G_bind_ and K_i_ (−10.03 kcal/mol and 0.04 µM, respectively), than examined protein-ligand complexes COX-1-IND (−9.64 kcal/mol and 0.08 µM, respectively). In the case of COX-2, the difference in calculated values of thermodynamic parameters was even more pronounced. In other words, the COX-2-CA protein-ligand complex attained significantly lower values of the ∆G_bind_ and K_i_ (−10.93 kcal/mol and 0.01 µM, respectively), than for examined protein-ligand complex COX-2-NS398 (−8.26 kcal/mol and 0.88 µM, respectively). It should be indicated that CA showed higher inhibitory potency against COX-2. Further analysis of the results presented defined CHR as the compound of interest for the inhibition of selected enzymes. In the case of protein-ligand complexes between COX-1 and CHR, very similar values of the ∆G_bind_ and K_i_ were accomplished as for the reference compound, IND (Table 5). Since the obtained values for CHR were slightly higher than the values for IND, it was acceptable to conclude that CHR could be considered a potential inhibitor of COX-1.

As regards the inhibition of COX-2 with CHR, based on the results presented in Table 6, it seems that CHR possessed better inhibitory potency against COX-2 than NS398, which was used as a reference compound, since lower values of ∆G_bind_ and K_i_ were achieved in molecular docking simulations with CHR (−9.51 kcal/mol and 0.12 µM, respectively) than with NS398 (−8.26 kcal/mol and 0.88 µM, respectively). Additionally, the higher calculated values of ∆G_bind_ and K_i_ for *p*-CA, FA, *p*-HBA, SIN, NAR, and RU implied that these compounds can bind to COX-1 and COX-2, but they can not be considered inhibitors of COX-1 and COX-2 (Table 5 and Table 6).

To explain the inhibitory potency of the tested compounds towards COX-1 and COX-2 enzymes, it is necessary to consider the structure of the targeted enzymes. A bundle of four amphipathic helices at the COX active site’s entry leads to a constriction made up of the residues Arg120, Tyr355, and Glu524. The primary cause of the significant variation in the isoforms’ active sites is the substitution of isoleucine from COX-1 at position 523 for valine in COX-2 [17]. The amino acids Gln192, His90, Leu517, Phe518, and Ile523 are defined as the side pocket region of the COX active site. It is observed that NS398 forms an ionic and hydrogen bond with the side chain of Arg120 at the opening of the cyclooxygenase channel, and a similar fact was detected for indomethacin [15].

A detailed examination of the contacts established in molecular docking simulations revealed that all tested compounds interacted with numerous amino acids from COX-1 and COX-2 and established different types of interactions. Among these interactions were hydrogen bonds, van der Waals, alkyl and π-alkyl, π-σ, π-Sulfur, π-Lone Pair, π-π Stacked, π-π-T-shaped, Amide- π-Stacked, Sulfur-X, and halogen interactions. It was noticed that three types of hydrogen bonds were present in the protein-ligand complex structures (Supplementary Figures S1–S6). One of them was the conventional hydrogen bond, and that is the most common type of hydrogen bond that is established. The second type of hydrogen bond was formed with carbon atoms and this type of bond is named the carbon-hydrogen bond. Also, the third type of hydrogen bond was observed, a π-donor hydrogen bond. This type of hydrogen bond is accomplished in molecular docking simulations between COX-2 and NAR and RU (Figure S6). The other recognized and mentioned interactions were from a group of hydrophobic contacts.

First, the interactions achieved in molecular docking simulations between COX-1 and tested compounds were discussed. IND was used as a reference compound and it established only two hydrogen bonds and numerous hydrophobic contacts (Supplementary Figure S1). A significantly lower number of contacts were established in simulations with CA, but it is interesting to point out that five hydrogen bonds were formed with interactions made with Arg120, Val349, Ser353, Tyr385, and Ser530. As regards interactions obtained from molecular docking simulations between COX-1 and CHR, six hydrogen bonds were observed with Tyr355, Ser530, Tyr385, Met522, and Leu384. Since CA and CHR had significant inhibitory potency compared with IND, it was interesting to observe that only CA interacted with Arg120, forming a hydrogen bond, whereas this bond was missing in simulations with IND and CHR. Other tested compounds formed various types of interactions with different amino acids from COX-1 (Supplementary Figures S1–S3).

Regarding the interactions obtained in molecular docking simulations with COX-2, the reference compound NS398 formed only two hydrogen bonds, one with Arg120 and one with Ser530 (Figure S4). Tyr355 formed one unfavorable acceptor-acceptor interaction in these simulations. The results presented in Table 6 imply that CA and CHR both possess higher inhibitory potency than NS398. Interactions presented in Supplementary Figures S4–S6 indicate that NS398 and CA formed a hydrogen bond with Arg120, unlike CHR. Notably, CHR established a lot of hydrophobic contacts and only three hydrogen bonds. It should be noticed that both NS398 and CHR formed unfavorable contacts, as opposed to CA.

In addition, in the case of all other compounds tested against COX-2, the formation of unfavorable interactions was noticed, except in the case of molecular docking simulations with *p*-CA and *p*-HBA (Supplementary Figures S4–S6).

## 3. Discussion

The content of phenolic compounds in the plant extracts is of immense importance since most often, phenolics are the bearers of biological activity and justify the medicinal application and consumption of the respective plant species. The class of polyphenolic compounds consists of various subgroups, such as flavonoids, phenolic acids, flavonols, tannins, etc. Most of these compounds exert quite important biological roles such as antioxidant, antiproliferative, antimicrobial, anti-inflammatory, anti-diabetic, and many other properties [18]. As this is one of the first studies on the chemical composition of *R. holostea* aerial parts, our findings showed, for the first time, a comprehensive analysis of polyphenolic derivatives, namely apigenin and luteolin C-glycosides, characteristic of the Caryophilaceae family.

Boulliant et al. [19], in one of the pioneer investigations of *R. holostea* chemical composition, also reported the isolation of apigenin C-glycosides, apigenin 6-C-(6-*O*-glucosylglucoside)-8-C-glucoside and 6-C-(6-*O*-glucosylglucoside)-8-C-glucoside. A more recent study by Ancheeva and coworkers [1], which evaluated and compared methanolic extracts from aerial parts of two species from the genus *Stellaria*, at that time, *S. nemorum* and *S. holostea* (*R. holostea*), showed the presence of luteolin-8-C-β-glucoside (orientin) and luteolin-6-C-β-glucoside (isoorientin) in *R. holostea* extract. Moreover, three flavonoids: apigenin 6-C-β-glucopyranoside-8-C-α-arabinopyranoside (schaftoside), diosmetin 6-C-β-glucopyranoside, and 3,5,7-trihydroxy-3′,5′-dimethoxyflavone, were also isolated from *R. holostea* in the same study. Besides these compounds, the detection and quantification of many others were reported in *R. holostea*. In the presented study, in the metabolic fingerprint several C-glucosides of apigenin and luteolin were identified, along with non-derivatized flavonoids apigenin and luteolin. Also, other flavonoid-type compounds were found, the flavone chrysoeriol and the flavanone eriodictyol. There exists some overlap with the results of the Mikšátková and collaborators’ study [20] that was focused on four different species from the genus *Stellaria* (*S. dichotoma*, *S. holostea*, *S. media*, and *S. nemorum*). All samples, including *R. holostea* (=*S. holostea*) had significant amounts of various flavonoids, such as apigenin and luteolin with their 7-*O*-glycosides, quercetin, rutin, kaempferol, and naringenin. By using high-performance liquid hromatography–electrospray ionization tandem mass spectrometry (HPLC– ESI-MS-MS) they quantified a significant amount of rutin in *R. holostea* (11.68 µg/g dry weight) several times higher than in the current study (3.4 µg/g). Luteolin and naringenin were also in higher quantity than in our tested sample, but in contrast to this study, we detected significant amounts of naringin and chrysoeriol. In addition, kaempferol was represented in a much larger quantity than in the aforementioned study. On the other hand, in contrast to Mikšátková and colleagues [20], in this study, isoflavonoids such as genistein, genistin, daidzin, and sophoricoside were not detected.

A recent study [21] focused on the determination of the phytochemical composition of the areal parts of three *Stellaria* spp., namely *S. bungeana, S. graminea,* and *S. holostea*. The obtained results showed that *S. holostea* (*R. holostea*) had a lower pectin concentration than the other two species, the amount of polysaccharides and hemicellulose was higher in *S. bungeana* than *S. holostea*, tannin concentration in *S. holostea* was higher than *S. bungeana* but lower than in *S. graminea,* and they all have a significant amount of vitamin C. Comparing all species it can be concluded that the lowest level of bioactive compounds was found in *S. holostea*, so it is not the primary choice for broader therapeutical use.

The literature sources are scarcely dealing with the evaluation of phenolic acids composition in *R. holostea*. Here, the presence of various groups of phenolic acids and their derivatives, from simple ones to hydroxycinnamic acids, was confirmed. The most recent review article by Jakimiuk et al. [22] gathered and counted all findings regarding the flavonoid compounds of *R. holostea* detected until now. As they showed, the largest amount of data regarding *R. holostea*, including those reported now, mostly overlap on jointly detected compounds, such as the largely glycosylated C- and *O*-derivatives of apigenin and luteolin, naringenin, kaempferol, chrysoeriol and rutin, all characteristic for the Caryophyllaceae family.

The available scientific data on the biological activity of *R. holostea* are also scarce. There is only some information on antimicrobial activity that showed that *R. holostea* aerial part methanolic extract was effective only against *Staphylococcus aureus*, unlike isolated C-glycosylflavonoids [1]. Similar results were reported for *S. media*. Its extract was effective both against *S. aureus* and *E. coli* [23]. Also, Kumarasamy et al. [24] reported the efficacy of *R. holostea* against *Pseudomonas aeruginosa* (MIC 0.1 mg/mL). In contrast, the antimicrobial activity of *R. holostea* extract in this study was mild to moderate (MIC 5–20 mg/mL). The antioxidant activity in four applied assays was at moderate levels. Although this extract contained a large number of phenolic compounds that are excellent antioxidants, it seems that they were not present in sufficient quantities to achieve this significant biological effect. In addition, it can be assumed that after the metabolism of C-glycosides of apigenin and luteolin in the body and their breakdown into aglycones, the antioxidant capacity would be significantly increased because these two flavonoids are considered exceptional antioxidants [25]. Similar results were reported for *S. media* aqueous and ethanolic extracts, where the ethanolic extract was more potent in free radicals’ scavenging [8]. Also, moderate values of antioxidant capacity considering ferric-reducing antioxidant power and ascorbic acid equivalent antioxidant capacity were reported for *S. media* ethanolic extract [23].

Of exceptional importance for this study is the confirmation of the traditional use of this plant species in the treatment of various inflammatory diseases. Until now, studies have not been carried out in this direction, so this is the first study that confirms a significant level of inhibition of two isoforms of cyclooxygenase that are important in the arachidonic acid pathway (COX-1 and COX-2) and play a key role in the synthesis of prostaglandins and thromboxanes. Reducing the concentration of these signaling molecules leads to the diminishing of inflammatory reactions [26]. Even though COX-2 activity is a dominant factor in the process of prostaglandin production during inflammation, there are some reports that the activities of both iso-forms are included in the acute inflammatory response [27]. The level of inhibition exhibited by *R. holostea* extract at the applied concentration in both cases was over 70%. These values were significantly higher in comparison with standard non-selective and selective inhibitors that were applied at defined concentrations. Moreover, *in silico* analysis confirmed that all compounds presented at the highest concentration in *R. holostea* extract showed inhibitory potential on both COX isoforms, where chlorogenic acid and chrysoeriol were particularly distinguished. The compelling inhibitory potential of chlorogenic acid [28] and chrysoeriol [29] against COX-1 and COX-2 enzymes were reported previously. Moreover, it should be pointed out that many other *R. holostea* constituents, which were not tested here, have immense anti-inflammatory properties. The following compounds stand out in particular: kaempferol, apigenin, and luteolin [25,30]; and phenolic acids such as rosmarinic acid, gallic acid, caffeic acid, *p*-coumaric acid, etc. [31,32,33]. Studies regarding the anti-inflammatory activity of *S. media* were not focused on the cyclooxygenase pathway, but they reported high inhibitory potential against xanthine oxidase, while simultaneously lowering the inhibition of pro-inflammatory enzymes hyaluronidase and lipoxidase activity [8]. The *S. media* extract was also able to launch the proliferation and migration of fibroblasts and act as a wound-healing agent *in vitro* [23].

## 4. Materials and Methods

### 4.1. Chemicals and Materials

UV–Vis double beam spectrophotometer Halo DB-20S (Dynamica GmbH, Dietikon, Switzerland) was used for all spectrophotometric measurements. The chemical and reagents used for the evaluation of phenolic contents and antioxidant activity were obtained from Alfa Aesar (Karlsruhe, Germany) and Sigma Aldrich (Steinheim, Germany). The solvents (methanol, acetonitrile, and formic acid) and reference standards of polyphenols (purity greater than 95%) for UHPLC analyses were purchased from Sigma Aldrich (Steinheim, Germany). All materials for antimicrobial activity tests (Nutrient agar—NA, Sabouraud dextrose agar—SDA, Müller–Hinton broth—MHB, and Sabouraud dextrose broth—SDB) were obtained from Torlak Institute of Virology, Vaccines and Sera (Belgrade, Serbia). Regarding the anti-inflammatory activity assays, the reagents and assay kits were purchased as follows: arachidonic acid, purified prostaglandin H synthase (PGHS)-1 from ram seminal vesicles, human recombinant COX-2, and NS-398 were purchased from Cayman Chemical Co. (Ann Arbor, MI, USA), Na_2_EDTA and tris(hydroxymethyl)-aminomethanhydro-chlorid (Titriplex III) from Merck KGaA (Darmstadt, Germany). Hematin and indomethacin (porcine) were obtained from ICN (Aurora, OH, USA), competitive PGE2 EIA kit from Enzo Life Sciences Inc. (Farmingdale, NY, USA), formic acid and DMSO (>99.98% purity) from Sigma-Aldrich (St. Louis, MO, USA), and epinephrine hydrogen tartarate from Fluka (St. Louis, MO, USA).

### 4.2. Plant Material and Extract Preparation

The plant material, aerial parts with flowers of *R. holostea* (Caryophyllaceae), was collected in April 2015, in the Ovčar-Kablar Gorge (Western Serbia) by J. S. Katanić Stanković. Prof. Dr. Milan S. Stanković conducted taxonomic and botanical identification and a voucher specimen (No. 123/015) was deposited in the Herbarium of the Department of Biology and Ecology, Faculty of Science, University of Kragujevac (Kragujevac, Serbia). The dried plant material (50 g) was shredded and powdered, followed by maceration with methanol (300 mL) at room temperature for 24 h. The maceration procedure was repeated three times, all the extracts were filtered and assembled followed by concentration using a rotary vacuum evaporator (RV 10 basic, IKA, Staufen, Germany) to obtain the dry extract. The percentage yield of *R. holostea* extract was 18.52% (*w*/*w*). The concentrations used in the experiments were calculated on the basis of the extract’s dry weight.

### 4.3. LC/MS Analysis

#### 4.3.1. UHPLC/MS-MS Orbitrap Analysis

Compounds of interest were separated on a Syncronis C18 column (100 × 2.1 mm, 1.7 μm particle size). The UHPLC Accela 600 system connected to LTQ Orbitrap MS hybrid mass spectrometer (Thermo Fisher Scientific, Bremen, Germany) was used for compounds identification. The all-chromatographic settings, heated electrospray ionization (HESI) and other MS parameters were previously as described [34].

Confirmation of some compounds was approved using available standards (see the header of Table 2), and the other compounds were identified according to HRMS and MS^n^ data with the consultation of previously published spectroscopic data on the analysis of secondary metabolites in *Stellaria* species [1,8,19,35,36] and generally in the family Caryophyllaceae [12,13,37,38,39].

#### 4.3.2. UHPLC/(-)HESI-MS^2^ Quantification of Major Phenolics

The extract of *R. holostea* was subjected to targeted metabolic profiling for the quantification of phenolic compounds. Analyses were performed using the Dionex Ultimate 3000 UHPLC system (Thermo Fisher Scientific, Bremen, Germany), configured with a triple quadrupole mass spectrometer (TSQ Quantum Access MAX, Thermo Fisher Scientific, Basel, Switzerland). Samples were chromatographically separated on a Synchronis aQ C18 column (100 × 2.1 mm) with 1.7 µm particle size (Thermo Fisher Scientific, Waltham, MA, USA), thermostated at 40 °C. Mobile phase, consisting of water + 0.1% formic acid (A) and acetonitrile + 0.1% formic acid (B), was eluted according to the gradient previously described by Banjanac et al. [40]. The flow rate of the mobile phase was set to 0.3 mL/min and the injection volume to 10 μL. A triple quadrupole mass spectrometer with heated electrospray ionization (HESI) was operated in a negative ionization mode, with the following parameter settings: vaporizer temperature 300 °C, spray voltage 4000 V, sheet gas (N_2_) pressure 26 AU, ion sweep gas (N_2_) pressure 1.0 AU and auxiliary gas (N_2_) pressure at 10 AU, capillary temperature 275 °C, skimmer offset 0 V. Argon was used as the collision gas in the collision-induced fragmentation of the molecules, and collision energy (cE) was set to 30 eV. Targeted compounds were quantified in a Single Reaction Monitoring (SRM) experiment by tracking two diagnostic MS^2^ fragments, and an external standard method was employed for the quantification. Calibration curves of targeted compounds showed excellent linearity with correlation coefficients r = 0.999, *p* < 0.001. Total concentrations of the analyzed phenolics were obtained by calculating the peak areas on MS chromatograms, and are expressed as mg per kg of dry extract (mg/kg d.e.). Xcalibur^TM^ software (version 2.2) was used for instrument control, data acquisition, and analysis.

### 4.4. Antioxidant Activity

#### 4.4.1. 2,2-Diphenyl-1-Picrylhydrazyl (DPPH) Free-Radical Scavenging Potential

The DPPH is a stable free radical often used to evaluate the antioxidant potential of various samples, such as plant extracts, pure, natural, or synthesized compounds, nanoparticles, etc. The method of Kumarasamy et al. [41] was used as we previously described [42]. The reference compounds used for the comparison of the results were caffeic acid, quercetin, and synthetic antioxidant butylated hydroxytoluene (BHT). The percentage of the radical scavenging activity was calculated as follows: % scavenging activity = [(A_c_ − A_s_)/A_c_] × 100, where A_c_ represents the absorbance of the control and A_s_ is the absorbance of the sample. The scavenging potency of tested samples towards DPPH free radical was expressed as IC_50_ values or, to be precise, the concentration of the sample that is able to reduce the concentration of free radicals to 50%. It was reported as µg/mL and calculated using a sigmoidal dose-response curve.

#### 4.4.2. 2,2′-Azinobis-(3-Ethylbenzothiazoline-6-Sulfonic Acid) Diammonium (ABTS) Radical-Cation Scavenging Potential

Another method frequently in use for determining the antioxidant activity is ABTS radical cation scavenging activity. The method was described in detail recently [42] according to Re et al. [43]. As in the previous method, caffeic acid, quercetin, and BHT were used as reference antioxidants. After calculating the percentage of ABTS^·+^ scavenging activity, as in the previous method, the IC_50_ values were evaluated as µg/mL, using a sigmoidal dose-response curve.

#### 4.4.3. Inhibition of Lipid Peroxidation

The method used for evaluating the level of inhibition of the lipid peroxidation process is the thiocyanate method [44] according to the already described procedure [42]. Linoleic acid emulsion was used as the source of lipids. The inhibition of the lipid peroxidation was calculated according to the equation: % inhibition = [(A_c_ − A_s_)/A_c_] × 100, where A_c_ represents the absorbance of the control and A_s_ is the absorbance of the sample. The IC_50_ values were expressed as µg/mL using a sigmoidal dose-response curve.

#### 4.4.4. Total Antioxidant Capacity

The total antioxidant capacity was determined using the method of Prieto et al. [45] which is based on the reduction of Mo(VI) from the reagent mixture to Mo(V), in the presence of antioxidants, in an acidic environment. The absorbance of the formed green phosphate-Mo(V) complex can be monitored at 695 nm. The full procedure was already reported [42]. Ascorbic acid was used as a standard antioxidant and total antioxidant capacity is expressed as ascorbic acid equivalents (mg AAE/g).

### 4.5. Antimicrobial Activity

#### 4.5.1. Tested Microorganisms

For the purpose of this experiment sixteen microorganism cultures (ATCC and isolated cultures) were used. They were obtained from the Laboratory for Microbiology, Department of Biology, Faculty of Science, University of Kragujevac, Kragujevac, Serbia and Institute of Public Health Kragujevac, University of Kragujevac, Serbia. Bacterial strains used were *Micrococcus lysodeikticus* ATCC 4698, *Enterococcus faecalis* ATCC 29212, *Escherichia coli* ATCC 25922, *Klebsiella pneumoniae* ATCC 70063, *Pseudomonas aeruginosa* ATCC 10145, *Bacillus mycoides* FSB 1, *Enterococcus faecalis* FSB 24, and *Azobacter chroococcum* FSB 14. Antifungal activity was evaluated using eight species: *Fusarium oxysporum* FSB 91, *Trichoderma longibrachiatum* FSB 13, *Penicillium verrucosum* FSB 21, *Penicillium canescens* FSB 24, *Aspergillus glaucus* FSB 32, *Alternaria alternata* FSB 51, *Phialophora fastigiata* FSB 81, and *Aspergillus brasiliensis* FSB 31. All microbial strains were kept under standard conditions (at 4 °C). They were subcultured as already described [42], particularly nutrient agar (NA) was used for maintaining bacteria, and fungi were grown in Sabouraud dextrose agar (SDA) and potato glucose agar (PDA), at adequate temperatures (37 and 28 °C, respectively) and time.

#### 4.5.2. Antimicrobial Activity Assays

The antibacterial and antifungal activities were evaluated using the microdilution method to define the minimum inhibitory concentration (MIC) of tested extract and reference compounds [46]. The assays were performed in 96 multi-well microtiter plates following CLSI [47] and NCCLS protocols [48,49]. As reference the compounds antibiotic chloramphenicol and antimycotic ketoconazole were used. All steps of analysis were followed as reported in Srećković et al. [50]. The lowest concentration with the absence of microorganism growth was defined as MIC.

### 4.6. Anti-Inflammatory Activity

#### 4.6.1. *In Vitro* Analysis of Anti-Inflammatory Activity

For the *in vitro* evaluation of the anti-inflammatory potential of the *R. holostea* extract, the assays used were based on the measurement of the inhibition of two crucial enzymes in the formation of prostaglandins in the arachidonic acid pathway, cyclooxygenase-1 (COX-1) and cyclooxygenase-2 (COX-2), according to manufacturer’s manual, as described by Fiebich et al. [51]. For COX-1 and -2 inhibition assays, the enzymes used were purified prostaglandin H synthase (PGHS)-1 from ram seminal vesicles and human recombinant PGHS-2, respectively. The assays were done in a 96-well plate format. The mixture for the incubation was comprised of TRIS/HCl-buffer (180 μL, 0.1 M, pH 8.0), 5 μM hematin, 18 mM epinephrine hydrogen tartrate, 0.2 U of enzyme, and the mixture for the COX-2 assay contained also 50 μM Na_2_EDTA. After incubation of the mixture for 5 min at room temperature, 10 μL of sample solutions were added. The extract (50 μg/mL) was dissolved in DMSO whereas ethanol was used to dissolve positive controls indomethacin (1.25 μM) and NS-398 (5 µM). By adding 10 μL of arachidonic acid (5 μM in ethanol) the reaction was started. The mixture was incubated at 37 °C and after 20 min 10 μL of 10% formic acid was added to stop the reaction. A competitive PGE_2_ EIA kit was used to evaluate the concentration of the main metabolite PGE_2_. The color was read using a microplate reader (Tecan). The inhibition of both enzymes’ activity was calculated in relation to a blank sample where an inhibitor was not added.

#### 4.6.2. *In Silico* Analysis of Anti-Inflammatory Activity

The inhibitory potencies of *p*-coumaric acid (*p*-CA), ferulic acid (FA), chlorogenic acid (CA), *p*-hydroxybenzoic acid (*p*-HBA), sinapic acid (SIN), chrysoeriol (CHR), naringin (NAR), and rutin (RU) towards COX-1 and COX-2 receptors were predicted *in silico*, using molecular docking simulations. For this purpose the AutoDock 4.2 software [52] was employed. The examined compounds were selected for examination since they were the dominant compounds in *R. holostea* extract, and they were used as ligands in the presented molecular docking simulations study. The preparation of selected compounds for molecular docking simulations was done by the optimization of their geometries in the gas phase using density functional theory (DFT). This was completed with Gaussian09 software [53], applying the b3lyp/6–311++g(d,p) theoretical model. The three-dimensional (3D) crystal structures of COX-1 and COX-2 enzymes were obtained from the Protein Data Bank (PDB IDs: 2OYU and 3QMO, respectively) [15,17]. The BIOVIA Discovery Studio 4.0 was used for the preparation of chosen enzymes to be set as receptors in molecular docking simulations [54]. The target receptors were rearranged by removing the co-crystallized ligands, water molecules, and cofactors. The binding sites of the target enzymes are recognized using AGFR (AutoGridFR) software [55], by detecting pockets and cavities of the known 3D structure of enzymes. The native-bound ligands were extracted from the structures of COX-1 and COX-2 enzymes and binding pockets analyses were accomplished. Further, re-docking was done with the selected compounds that are used as ligands and the same docking modes were generated as found in co-crystallized forms of target enzymes. For the addition of polar hydrogen atoms and the calculation of Kollman charges, the AutoDockTools (ADT) graphical user interface was used. The bonds in the ligands are set to be rotatable and the ligands are set to be flexible. The structure of target enzymes remains standing as rigid. The Lamarckian Genetic Algorithm (LGA) was used for enzyme-ligand flexible molecular docking simulations. The grid boxes centers with dimensions 21.303 Å × 50.860 Å × 13.585 Å and 40.250 Å × 50.324 Å × 67.546 Å in -x, -y, and -z directions of COX-1 and COX-2 were used to cover the enzymes binding sites and accommodate ligands to move spontaneously. A grid point spacing of 0.375 Å was used for auto grid runs. All the molecular docking simulations were performed at a temperature of 298.15 K. Analysis of molecular docking simulation results and visualizations were accomplished using BIOVIA Discovery Studio.

For the valuation of the inhibitory potency, AutoDock uses empirical scoring functions established on the free energy of binding (∆G_bind_). The value of ∆G_bind_ depends on the values of Final Intermolecular Energy (FIE), Final Total Internal Energy (FTIE), Torsional Free Energy (TFE), and Unbound System’s Energy (USE) (Equation (1)). The value of FIE is a summary of the Van der Waals energy, energy of hydrogen bonds, desolvation energy of the system, and electrostatic energy. The energy that is released during the creation of contacts between a ligand and a target protein is represented by the value of ∆G_bind_.

∆G_bind_ = [(FIE) + (FTIE) + (TFE)-(USE)] (1)

For the prediction of the inhibitory potency of some compounds, there is one more important parameter and that is the inhibitory constant (K_i_). This value is calculated by AutoDock after the estimation of the free energy of binding. The inhibitory constant value is determined by the following equation:

K_i_ = exp(∆G_bind_/RT) (2)

where R is the gas constant (R = 1.99 cal/molK), and T is the value of the room temperature (298.15 K).

### 4.7. Statistical Analysis

The data were expressed as mean ± S.D. Statistical evaluation of the data was performed by one-way analysis of variance (ANOVA) using the Origin 2019b statistical software package, for Windows. The results were considered statistically significant at *p* < 0.05.

## 5. Conclusions

The presented study reported the phytochemical composition of *R. holostea* methanolic extract and quantified a number of flavonoids, phenolic acids, and their derivatives. It was shown that the extract was quite rich in apigenin and luteolin C-glycosyl-flavonoids, characteristic for the Caryophyllaceae family. Besides, verbascoside and chrysoeriol were identified in this plant species for the first time. *R. holostea* aerial part was rich in phenolic acids, particularly in *p*-coumaric, chlorogenic, and ferulic acids. The most abundant in the group of flavonoids were naringin, chrysoeriol, and rutin. Total concentrations of phenolic compounds were directly related to moderate biological activity, in terms of *in vitro* antioxidant and antimicrobial properties. Nevertheless, *in vitro* anti-inflammatory effects of *R. holostea* methanolic extract against COX-1 and COX-2 activities were quite pronounced. The compounds present in the largest quantities that contributed the most to this activity were chlorogenic acid and chrysoeriol, as confirmed by *in silico* tests. The obtained results showed, for the first time, the justification of the traditional application of the investigated plant species and encouraged further research in this direction. Of particular importance will be the detailed description of the mechanism of anti-inflammatory action, the behavior of the extract in *in vivo* conditions, the impact of the gastrointestinal microbiota, and finding new ways and forms of application of *R. holostea*.

## Figures and Tables

**Table 1:** UHPLC–MS^4^ Orbitrap metabolic fingerprint (negative ionization mode) of *R. holostea* extract.

No	Compound Name	*t*_R_, min	Molecular Formula, [M–H]^−^	Calculated Mass, [M–H]^−^	Exact Mass, [M–H]^−^	Δ mDa	MS^2^ Fragments, (% Base Peak)	MS^3^ Fragments, (% Base Peak)	MS^4^ Fragments, (% Base Peak)
***Hydroxybenzoic acids***									
**1**	**Gallic acid*^a^***	4.69	C_7_H_5_O_5_^−^	169.01425	169.01217	2.08	69(5), 84(7), 123(8), 124(7), 125(100), 126(8), 127(3)	53(58), 81(100), 83(6), 97(85), 98(21), 125(9)	ND
**2**	**Dihydroxybenzoic acid hexoside I**	5.65	C_13_H_15_O_9_^−^	315.07216	315.06992	2.23	108(8), 109(12), 152(50), 153(100), 163(9), 165(12), 268(8)	109(100), 123(3)	ND
**3**	**Dihydroxybenzoic acid hexoside II**	5.97	C_13_H_15_O_9_^−^	315.07216	315.07002	2.14	109(9), 135(3), 151(4), 153(100), 154(6)	109(100), 123(6)	53(18), 81(100)
**4**	***p*-Hydroxybenzoic acid*^a^***	6.70	C_7_H_5_O_3_^−^	137.02442	137.02354	0.88	93(100)	ND	ND
***Hydrocinnamic acids***									
**5**	**Coumaroylquinic acid I**	6.20	C_16_H_17_O_8_^−^	337.09289	337.09048	2.42	119(21), 145(100), 163(56), 219(20), 277(50), 293(17), 319(35)	117(100), 145(3)	ND
**6**	**Coumaric acid dihexoside**	6.52	C_21_H_27_O_13_^−^	487.14572	487.14282	2.90	145(7), 163(100), 187(20), 221(4), 323(14), 397(5), 427(8)	119(100)	ND
**7**	**Coumaroylquinic acid II**	6.81	C_16_H_17_O_8_^−^	337.09289	337.09043	2.47	117(6), 119(16), 145(100), 146(8), 163(58), 277(52), 291(6)	117(100)	ND
**8**	**5-*O*-Caffeoylquinic acid (Chlorogenic acid)*^a^***	6.95	C_16_H_17_O_9_^−^	353.08781	353.08502	2.79	179(3), 191(100), 192(6)	85(100), 87(19), 111(33), 127(83), 171(24), 173(57)	53(100)
**9**	**Caffeic acid*^a^***	7.76	C_9_H_7_O_4_^−^	179.03498	179.03400	0.98	89(23), 133(24), 134(12), 135(100), 136(14), 143(17), 161(18)	78(7), 91(27), 93(6), 106(19), 107(100)	ND
**10**	**Verbascoside**	8.12	C_29_H_35_O_15_^−^	623.19814	623.19537	2.78	315(3), 461(100), 462(14)	135(66), 143(6), 161(13), 297(16), 315(100)	119(11), 135(100), 143(4), 161(3), 179(3)
**11**	***p*-Coumaric acid*^a^***	8.57	C_9_H_7_O_3_^−^	163.04007	163.03920	0.87	119(100), 120(8), 121(5), 131(6), 133(5), 135(6), 136(4)	91(100), 92(11), 168(9)	ND
**12**	**Sinapic acid*^a^***	9.02	C_11_H_11_O_5_^−^	223.06120	223.05900	2.20	164(18), 179(31), 208(100)	149(13), 164(100), 193(9)	135(34), 149(100)
**13**	**Ferulic acid*^a^***	9.14	C_10_H_9_O_4_^−^	193.05063	193.04956	1.08	111(57), 134(34), 147(100), 148(10), 149(95), 150(10), 178(71)	57(4), 85(6), 99(4), 103(100), 119(3), 129(41)	59(100)
**14**	**Rosmarinic acid**	9.22	C_18_H_15_O_8_^−^	359.07517	359.07724	−2.07	161(100), 297(63), 313(40), 341(26), 197(23)	133(100)	ND
**15**	**Coumaric acid methyl ester**	11.32	C_10_H_9_O_3_^−^	177.05572	177.05467	1.05	117(13), 118(43), 119(3), 145(100), 146(9), 162(36), 177(8)	83(3), 117(100)	ND
***Flavonoid C-glycosides***									
**16**	**Luteolin 6-*C*-pentoside-8-*C*-(6”-hexosyl)-hexoside**	6.45	C_32_H_37_O_20_^−^	741.18249	741.18096	1.53	369(22), 399(34), 429(11), 441(11), 459(100), 460(19), 489(38)	369(100), 381(4), 399(88), 423(3), 441(30)	298(31), 312(4), 313(40), 341(100)
**17**	**Apigenin 6-*C*-hexoside-8-*C*-(6”-hexosyl)-hexoside**	6.52	C_33_H_39_O_20_^−^	755.19814	755.19914	−1.00	353(71), 354(14), 383(42), 473(100), 474(19), 635(34), 665(23)	353(100), 354(5), 383(31), 455(5)	282(3), 297(53), 307(3), 325(100), 326(3)
**18**	**Apigenin 6-*C*-(6”-hexosyl)-hexoside 8-*C*-pentoside I**	6.70	C_32_H_37_O_19_^−^	725.18758	725.18392	3.66	353(64), 383(60), 443(100), 444(19), 635(34), 665(31), 707(15)	353(100), 354(6), 365(5), 383(55), 384(3), 425(18)	233(3), 297(54), 325(100), 335(3)
**19**	**Luteolin 8-*C*-(6”-hexosyl)-hexoside**	6.95	C_27_H_29_O_16_^−^	609.14024	609.14037	−0.14	297(6), 327(100), 328(12), 357(100), 358(14), 369(12), 393(7)	133(3), 191(3), 255(4), 284(20), 299(100), 300(11)	213(55), 231(27), 240(39), 255(100), 257(26)
**20**	**Apigenin 6-*C*-(6”-hexosyl)-hexoside 8-*C*-pentoside II**	6.98	C_32_H_37_O_19_^−^	725.19345	725.19138	2.07	353(47), 383(30), 443(100), 444(19), 473(38), 527(16), 635(18)	353(100), 354(5), 383(26), 425(3)	282(3), 297(49), 325(100), 326(3)
**21**	**Apigenin 6,8-di-*C*-hexoside**	7.11	C_27_H_29_O_15_^−^	593.15119	593.14703	4.16	353(44), 354(10), 383(23), 473(100), 474(20), 503(30), 575(9)	353(100), 354(4), 383(16)	282(3), 297(53), 325(100)
**22**	**Luteolin 6-*C*-pentoside-8-*C*-hexoside**	7.18	C_26_H_27_O_15_^−^	579.12967	579.13175	−2.08	369(21), 399(30), 459(53), 489(100), 490(20), 519(19), 561(14)	369(100), 370(5), 399(67), 411(4), 429(17), 471(12)	298(28), 312(5), 313(33), 341(100)
**23**	**Apigenin 8-*C*-(6”-hexosyl)-hexoside**	7.27	C_27_H_29_O_15_^−^	593.15119	593.14849	2.71	246(3), 283(11), 311(100), 312(10), 341(19), 353(4), 473(7)	283(100), 284(10)	163(88), 211(30), 224(28), 239(100), 283(50)
**24**	**Apigenin 6-*C*-hexoside 8-*C*-pentoside**	7.46	C_26_H_27_O_14_^−^	563.14063	563.13740	3.23	353(29), 383(22), 443(100), 444(21), 473(59), 474(14), 545(11)	353(100), 354(13), 383(23), 384(3), 425(3)	297(47), 298(4), 323(3), 325(100)
**25**	**Luteolin 8-*C*-hexoside**	7.78	C_21_H_19_O_11_^−^	447.09329	447.09015	3.13	172(3), 327(100), 328(8), 357(48), 358(3), 369(3), 429(10)	284(9), 298(3), 299(100), 300(7)	199(33), 213(65), 231(33), 240(46), 255(100)
**26**	**Chrysoeriol (3′-Methyl luteolin) 6-C-hexoside**	8.36	C_22_H_21_O_11_^−^	461.10894	461.10556	3.37	341(100), 342(8), 371(16), 443(3)	298(100), 313(29), 326(5)	253(49), 255(38), 269(88), 270(100), 298(94)
**27**	**Luteolin 6-*C*-hexoside-8-*C*-(2”-coumaroyl)-hexoside**	8.57	C_36_H_35_O_18_^−^	755.18289	755.18424	−1.35	297(10), 327(100), 328(14), 357(67), 358(11), 369(10), 609(12)	255(3), 284(18), 285(3), 298(5), 299(100), 300(9)	213(62), 227(43), 240(46), 255(100), 257(40)
***Flavonoid O-glycosides***									
**28**	**Quercetin 3-*O*-(6”-rhamnosyl)-hexoside (Rutin)*^a^***	8.00	C_27_H_29_O_16_^−^	609.14611	609.14506	1.05	255(5), 271(8), 285(5), 300(41), 301(100), 302(17), 343(8)	151(66), 179(100), 229(6), 256(13), 272(15), 273(19)	151(100)
**29**	**Quercetin 3-*O*-galactoside (Hyperoside)*^a^***	8.50	C_21_H_19_O_12_^−^	463.08233	463.08276	−0.44	299(3), 300(22), 301(100), 302(15)	151(81), 179(100), 255(12), 257(14), 271(19), 273(18)	151(100)
**30**	**Naringin 7-*O*-(2”-rhamnosyl)-hexoside (Naringin)*^a^***	8.85	C_27_H_31_O_14_^−^	579.16606	579.16645	−0.39	235(13), 271(48), 272(7), 313(17), 357(5), 459(100), 460(17)	151(22), 235(68), 271(49), 339(28), 357(100), 441(23)	125(14), 151(79), 168(24), 169(17), 339(100)
**31**	**Jaceosidin 7-*O*-hexoside (Jaceoside)**	8.99	C_23_H_23_O_12_^−^	491.11363	491.11550	−1.87	314(13), 328(15), 329(58), 330(8), 343(9), 476(100), 477(23)	313(38), 314(99), 315(33), 343(100), 357(10), 461(63)	315(32), 328(100), 329(4)
**32**	**Jaceosidin 7-*O*-hexuronide**	9.08	C_23_H_21_O_13_^−^	505.09877	505.09558	3.18	175(4), 315(4), 329(100), 330(15)	299(4), 314(100), 315(9)	285(9), 299(100)
***Flavonoid aglycones***									
**33**	**Catechin*^a^***	7.22	C_15_H_13_O_6_^−^	289.07176	289.06838	3.38	179(12), 203(12), 205(37), 231(7), 245(100), 246(16), 247(8)	161(18), 187(24), 188(14), 203(100), 227(25), 230(5)	161(53), 173(15), 175(100), 185(35), 188(77)
**34**	**Eriodictyol *^a^***	10.63	C_15_H_11_O_6_^−^	287.05024	287.05213	−1.90	151(100), 152(8), 199(8), 241(9), 253(5), 257(8), 269(4)	65(4), 83(5), 107(100)	65(100)
**35**	**Luteolin *^a^***	10.71	C_15_H_9_O_6_^−^	285.04046	285.03692	3.54	151(37), 175(79), 199(80), 217(65), 241(100), 243(53), 285(45)	197(81), 198(100), 199(51), 212(18), 213(39), 226(31)	ND
**36**	**Naringenin *^a^***	11.57	C_15_H_11_O_5_^−^	271.06120	271.05761	3.59	107(5), 149(8), 151(100), 152(7), 177(21), 225(19), 227(5)	65(4), 83(5), 107(100)	65(100)
**37**	**Apigenin *^a^***	11.61	C_15_H_9_O_5_^−^	269.04950	269.04810	1.39	149(38), 151(100), 181(18), 201(27), 225(93), 227(20), 269(34)	83(3), 107(100)	63(12), 65(100)
**38**	**Kaempferol *^a^***	11.78	C_15_H_9_O_6_^−^	285.04046	285.03693	3.53	229(15), 241(16), 255(67), 256(100), 257(45), 284(19), 285(46)	211(36), 212(71), 227(100), 228(51), 229(20), 256(17)	ND
**39**	**Chrysoeriol *^a^***	11.82	C_16_H_11_O_6_^−^	299.05024	299.05222	−1.98	284(100), 285(13)	256(100), 284(6)	211(17), 212(16), 227(100), 228(33), 239(15)

*^a^* Confirmed using reference standards; the other compounds were identified based on HRMS data and MS^n^; *t*_R_—retention time (min); Δ ppm—mean mass accuracy; “ND”—not detected.

**Table 2:** UHPLC/(−)HESI–MS/MS quantitative method and data of targeted phenolic compounds in *R. holostea* extract. Concentration is presented as mg per kg of the dry extract [mg/kg d.e.]. Values are means of three replicates ± SD.

Phenolic Compound	t_R_, min	Linearity Equations (A + BX) × 10^5^	Correlation *R*^2^	LOD, µg/mL	LOQ, µg/mL	Parent Ion, m/z	Product Ion, m/z (Collision Energy, eV)	Content (mg/kg d.e.)
Gallic acid	2.14	Y = −0.23 + 5.19X	0.9905	0.12	0.41	169.032	79.11 (31); 125.04 (16)	1.04
Chlorogenic acid	4.99	Y = −0.48 + 34.94X	0.9923	0.13	0.43	353.103	191.28 (25)	46.35
*p*-Hydroxybenzoic acid	5.23	Y = −0.25 + 2.70X	0.9916	0.21	0.70	137.057	93.19 (19); 108.33 (22)	6.54
Catechin	5.46	Y = −0.11 + 3.23X	0.9937	0.08	0.26	289.050	245.10 (16); 123.08 (34)	0.47
Caffeic acid	5.51	Y = −1.18 + 55.82X	0.9917	0.11	0.38	179.004	134.00 (13); 135.00 (16)	2.09
Rutin	6.04	Y = 0.63 + 38.76X	0.9939	0.14	0.46	609.197	299.98 (42); 301.20 (32)	3.41
*p*-Coumaric acid	6.15	Y = −0.41 + 36.64X	0.9923	0.10	0.33	163.031	93.12 (39); 119.09 (16)	81.18
Hyperoside	6.40	Y = 0.63 + 60.91X	0.9927	0.10	0.32	463.002	271.01 (44); 300.02 (29)	0.84
Ferulic acid	6.55	Y = 0.08 + 10.07X	0.9978	0.04	0.13	193.057	134.00 (18); 178.00 (15)	42.98
Sinapic acid	6.68	Y = −0.02 + 0.69X	0.9941	0.09	0.31	223.082	149.21 (36)	4.20
Naringin	6.84	Y = 0.02 + 1.41X	0.9915	0.11	0.35	579.241	271.36 (33); 151.42 (43)	1.16
Eriodictyol	8.12	Y = −0.87 + 38.38X	0.9974	0.06	0.19	286.974	150.93 (19); 135.02 (22)	0.34
Luteolin	8.21	Y = −2.09 + 54.69X	0.9977	0.05	0.17	285.035	151.03 (18); 133.06 (36)	0.35
Naringenin	8.88	Y = −0.54 + 36.66X	0.9990	0.04	0.12	271.036	151.01 (20); 107.07 (26)	0.15
Apigenin	8.89	Y = −1.16 + 45.10X	0.9973	0.06	0.20	269.032	151.00 (26); 117.07 (43)	0.18
Kaempferol	8.91	Y = −0.08 + 2.64X	0.9913	0.12	0.39	285.074	211.00 (32); 227.00 (32)	0.86
Chrysoeriol	9.15	Y = −0.40 + 7.19X	0.9946	0.07	0.24	298.933	210.89 (43); 159.17 (26)	3.83

**Table 3:** Antioxidant activity of *R. holostea* aerial part extract.

Sample and Standards	IC_50_ Values (μg/mL)			Total Antioxidant Capacity (mg AAE/g)
	**DPPH**· **Scavenging Activity**	**ABTS**^·+^ **Scavenging Activity**	Inhibition of Lipid Peroxidation	
*R. holostea* extract	246.7 ± 6.8 ^c^	420.4 ± 9.3 ^b^	570.4 ± 9.9	192.0 ± 3.9
CA	2.97 ± 0.31 ^a^	12.16 ± 2.04 ^a^	-	-
QU	1.41 ± 0.19 ^a^	8.37 ± 1.12 ^a^	-	-
BHT	13.61 ± 1.74 ^b^	26.09 ± 2.84 ^a^	3.92 ± 0.76	-

Data are represented as mean ± SD (n = 3). IC_50_ values were determined by nonlinear regression analysis; AAE—ascorbic acid equivalents; CA—caffeic acid; QU—quercetin; BHT—butylated hydroxytoluene. Means in the same column with different letters as superscripts are significantly different at *p* < 0.05.

**Table 4:** Antimicrobial properties of *R. holostea* aerial part methanol extract.

Bacteria (ATCC and Isolates)	MIC Values		Fungi	MIC Values	
	*R. holostea* Extract	Chloramphenicol		*R. holostea* Extract	Ketoconazole
*Micrococcus lysodeikticus* ATCC 4698, G+	10 × 10^3^	2.5	*Fusarium oxysporum* FSB 91	1250	0.31
*Enterococcus faecalis* ATCC 29212, G+	10 × 10^3^	10	*Trichoderma longibrachiatum* FSB 13	20 × 10^3^	1.25
*Enterococcus faecalis* FSB 24, G+	5 × 10^3^	2.5	*Phialophora fastigiata* FSB 81	5 × 10^3^	10
*Bacillus mycoides* FSB 1, G+	20 × 10^3^	10	*Alternaria alternata* FSB 51	10 × 10^3^	5
*Escherichia coli* ATCC 25922, G-	10 × 10^3^	10	*Penicillium verrucosum* FSB 21	10 × 10^3^	2.5
*Klebsiella pneumoniae* ATCC 70063, G-	10 × 10^3^	10	*Penicillium canescens* FSB 24	5 × 10^3^	1.25
*Pseudomonas aeruginosa* ATCC 10145, G-	>20 × 10^3^	10	*Aspergillus glaucus* FSB 32	20 × 10^3^	2.5
*Azobacter chroococcum* FSB 14, G-	5 × 10^3^	5	*Aspergillus brasiliensis* FSB 31	20 × 10^3^	0.62

MIC, minimum inhibitory concentration values are given as μg/mL.

**Table 5:** Important thermodynamical parameters from docking simulations between indomethacin (IND), selected compounds *p*-coumaric acid (*p*-CA), ferulic acid (FA), chlorogenic acid (CA), *p*-hydroxybenzoic acid (*p*-HBA), sinapic acid (SIN), chrysoeriol (CHR), naringin (NAR), and rutin (RU) with COX-1.

Complexes	∆G_bind_(kcal/mol)	**K_i_ (µM)**	FIE (kcal/mol)	vdW + Hbond + Desolv Energy (kcal/mol)	Electrostatic Energy (kcal/mol)	FTIE (kcal/mol)	TFE (kcal/mol)	USE (kcal/mol)
**COX-1-IND**	−9.64	0.08	−11.31	−10.25	−1.06	−0.53	+1.49	−0.71
**COX-1-*p*-CA**	−5.46	99.17	−6.77	−5.89	−0.88	+0.05	+1.19	−0.06
**COX-1-** **FA**	−6.00	40.21	−6.89	−6.05	−0.83	−0.84	+1.49	−0.24
**COX-1-** **CA**	−10.03	0.04	−9.75	−8.79	−0.96	−4.93	+3.28	−1.37
**COX-1-*p*-HBA**	−4.94	239.52	−5.84	−5.59	−0.25	−0.01	+0.89	−0.01
**COX-1-SIN**	−6.32	23.24	−7.57	−6.95	−0.62	−1.12	+1.79	−0.58
**COX-1-** **CHR**	−9.36	0.14	−9.62	−9.49	−0.13	−1.63	+1.49	−0.40
**COX-1-** **NAR**	−4.66	382.65	−4.37	−4.31	−0.07	−6.55	+4.18	−2.09
**COX-1-** **RU**	−6.41	20.04	−8.16	−7.99	−0.17	−5.20	+4.77	−2.17

**Table 6:** Important thermodynamical parameters from docking simulations between NS398, selected compounds *p*-coumaric acid (*p*-CA), ferulic acid (FA), chlorogenic acid (CA), *p*-hydroxybenzoic acid (*p*-HBA), sinapic acid (SIN), chrysoeriol (CHR), naringin (NAR), and rutin (RU) with COX-2.

Complexes	∆G_bind_ (kcal/mol)	**K_i_ (µM)**	FIE (kcal/mol)	vdW + Hbond + Desolv Energy (kcal/mol)	Electrostatic Energy (kcal/mol)	FTIE (kcal/mol)	TFE (kcal/mol)	USE (kcal/mol)
**COX-2-NS398**	−8.26	0.88	−9.75	−9.08	−0.67	−0.94	+1.49	−0.93
**COX-2-*p*-CA**	−5.08	188.97	−6.28	−5.40	−0.88	−0.06	+1.19	−0.06
**COX-2-** **FA**	−5.96	42.42	−6.90	−6.20	−0.70	−0.80	+1.49	−0.24
**COX-2-** **CA**	−10.93	0.01	−11.52	−11.04	−0.49	−4.06	+3.28	−1.37
**COX-2-*p*-HBA**	−4.52	488.92	−5.41	−4.50	−0.91	−0.01	+0.89	−0.01
**COX-2-SIN**	−6.21	28.03	−7.47	−6.77	−0.70	−1.11	+1.79	−0.58
**COX-2-** **CHR**	−9.51	0.12	−9.79	−9.73	−0.06	−1.61	+1.49	−0.40
**COX-2-** **NAR**	−5.53	88.88	−6.57	−6.62	+0.04	−5.23	+4.18	−2.10
**COX-2-** **RU**	−4.13	937.71	−6.74	−6.72	−0.02	−4.36	+4.77	−2.20

## Data Availability

Not applicable.

## Supplementary Materials

The following supporting information can be downloaded at: https://www.mdpi.com/article/10.3390/molecules28031274/s1, Figure S1. The established interactions of the most stable conformations of protein-ligand complex structures of: A—indomethacin (IND), B—*p*-coumaric acid (*p*-CA), and C—ferulic acid (FA) and COX-1; Figure S2. The established interactions of the most stable conformations of protein-ligand complex structures of: A—chlorogenic acid (CA), B—*p*-hydroxybenzoic acid (*p*-HBA), and C—sinapic acid (SYN) and COX-1; Figure S3. The established interactions of the most stable conformations of protein-ligand complex structures of: A—chrysoeriol (CHR), B—naringin (NAR), and C—rutin (RU) and COX-1; Figure S4. The established interactions of the most stable conformations of protein-ligand complex structures of: A—NS398, B—*p*-coumaric acid (*p*-CA), and C—ferulic acid (FA) and COX-2; Figure S5. The established interactions of the most stable conformations of protein-ligand complex structures of: A—chlorogenic acid (CA), B—*p*-hydroxybenzoic acid (*p*-HBA), and C—sinapic acid (SYN) and COX-2; Figure S6. The established interactions of the most stable conformations of protein-ligand complex structures of: A—chrysoeriol (CHR), B—naringin (NAR), and C—rutin (RU) and COX-2.

## Sample Availability

Samples of the compounds are not available from the authors upon request.
