# Supplementary material for: UHPLC-MS Phytochemical Profiling and Insight into Bioactivity of Rabelera holostea (Greater Stitchwort) Extract
Source: Molecules. 2023 Jan 28;28(3):1274. doi: 10.3390/molecules28031274 (PMC9921532; doi:10.3390/molecules28031274)
Supplement: Supplementary file 1 [file molecules-28-01274-s001.zip › molecules-2157459-supplementary.pdf]

Supplementary material

UHPLC-MS Phytochemical Profiling and Insight into Bioactivity of Rabelera holostea (Greater Stitchwort) Extract

Jelena S. Katanić Stanković [1,*], Jelena Đorović Jovanović [1], Danijela Mišić [2], Uroš Gašić [2], Stefanie Nikles [3], Zoran Marković [1], Rudolf Bauer [3,*]

[1] *University of Kragujevac, Institute for Information Technologies Kragujevac, Department of Science, Jovana Cvijića bb, 34000 Kragujevac, Serbia*

[2] *University of Belgrade, Institute for Biological Research "Siniša Stanković" – National Institute of Republic of Serbia, Department of Plant Physiology, Bulevar Despota Stefana 142, 11060 Belgrade, Serbia*

[3] *University of Graz, Institute for Pharmaceutical Sciences, Department of Pharmacognosy, Bethovenstrasse 8, 8010 Graz, Austria*

Correspondence: jkatanic@kg.ac.rs (J.S.K.S.); rudolf.bauer@uni-graz.at (R.B.)

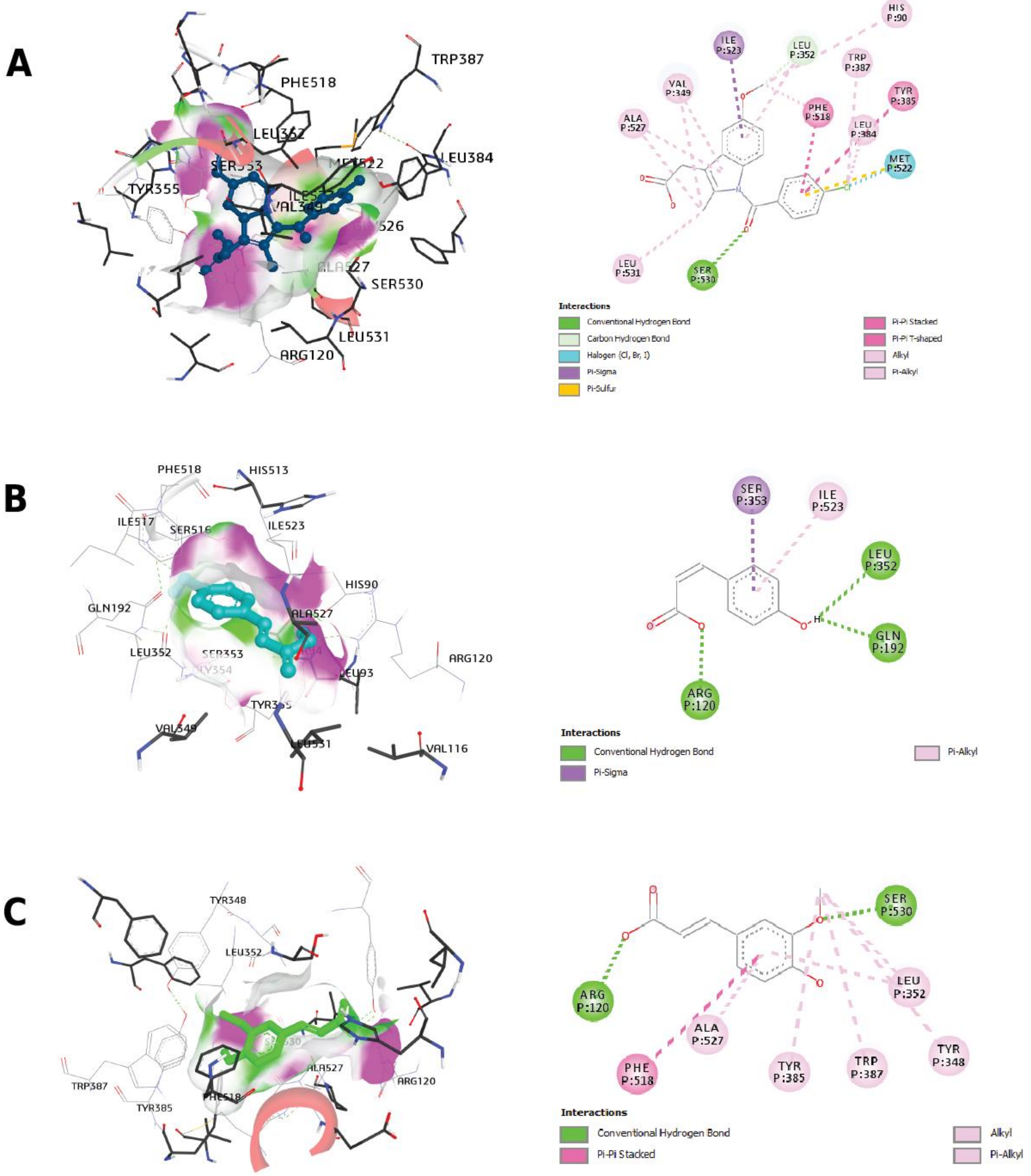

Figure S1. The established interactions of the most stable conformations of protein-ligand complex structures of: A – indomethacin (IND), B – *p*-coumaric acid (*p*-CA), and C – ferulic acid (FA) and COX-1.

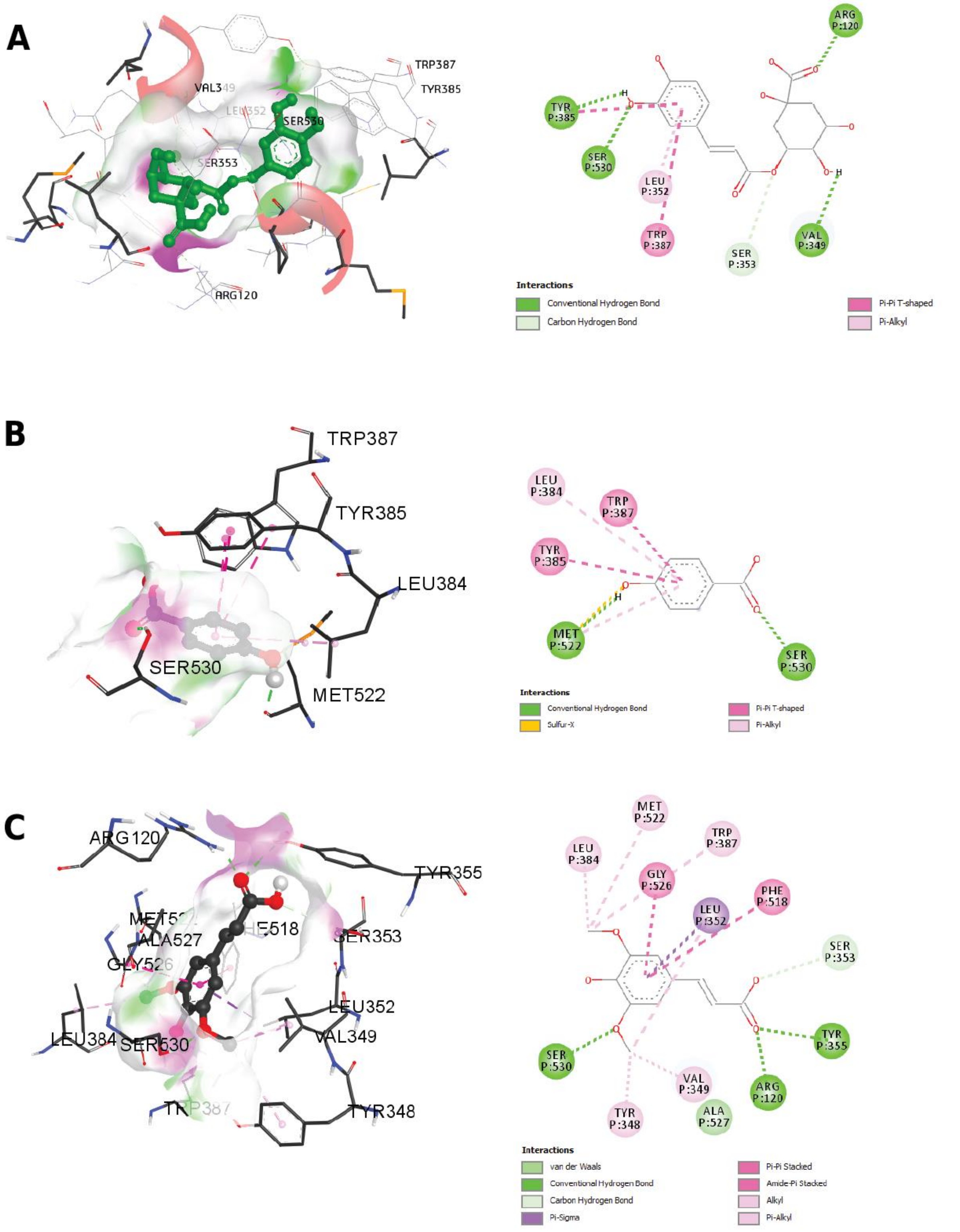

Figure S2. The established interactions of the most stable conformations of protein-ligand complex structures of: A – chlorogenic acid (CA), B – *p*-hydroxybenzoic acid (*p*-HBA), and C – synapic acid (SYN) and COX-1.

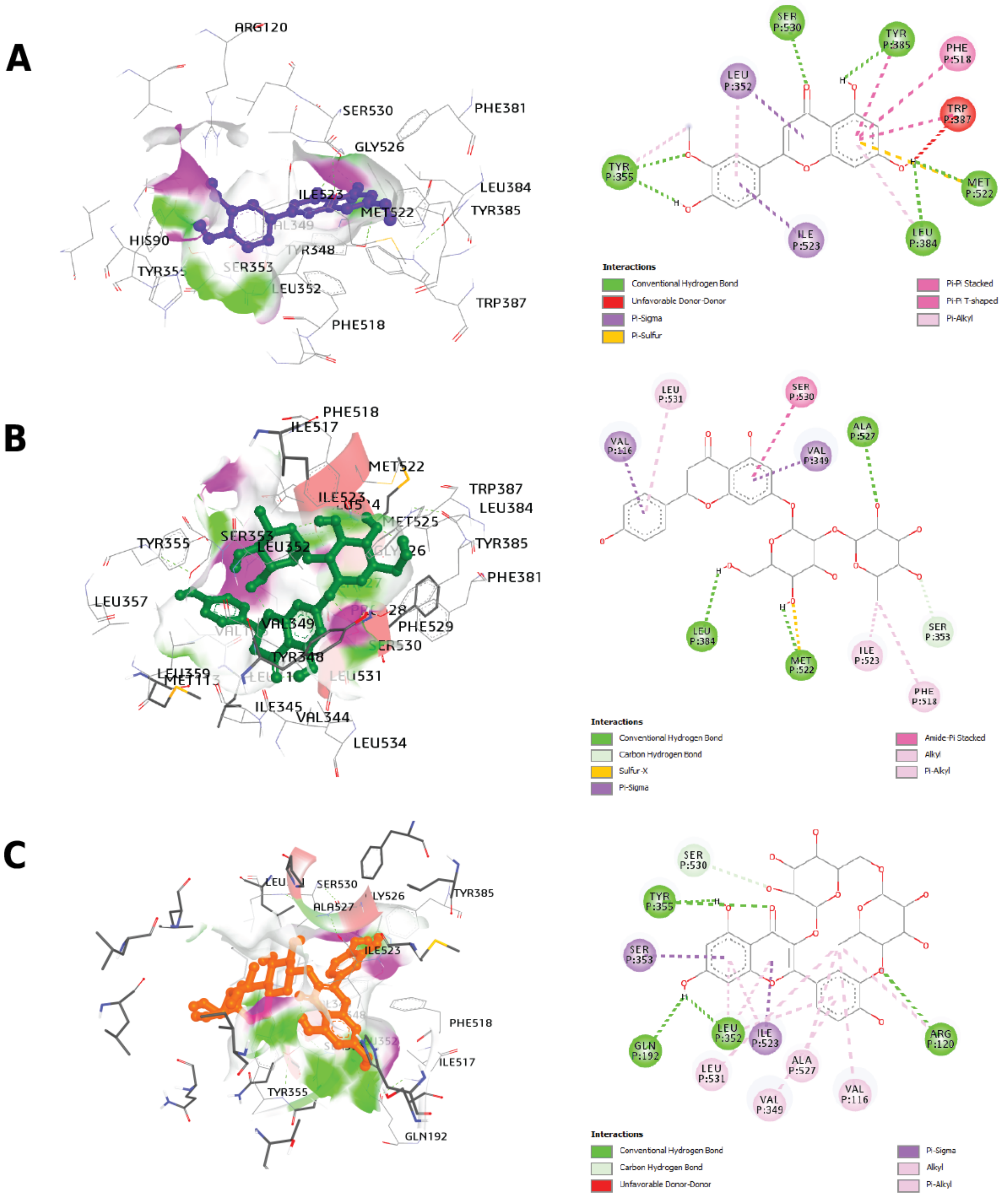

Figure S3. The established interactions of the most stable conformations of protein-ligand complex structures of: A – chrysoeriol (CHR), B – naringin (NAR), and C – rutin (RU) and COX-1.

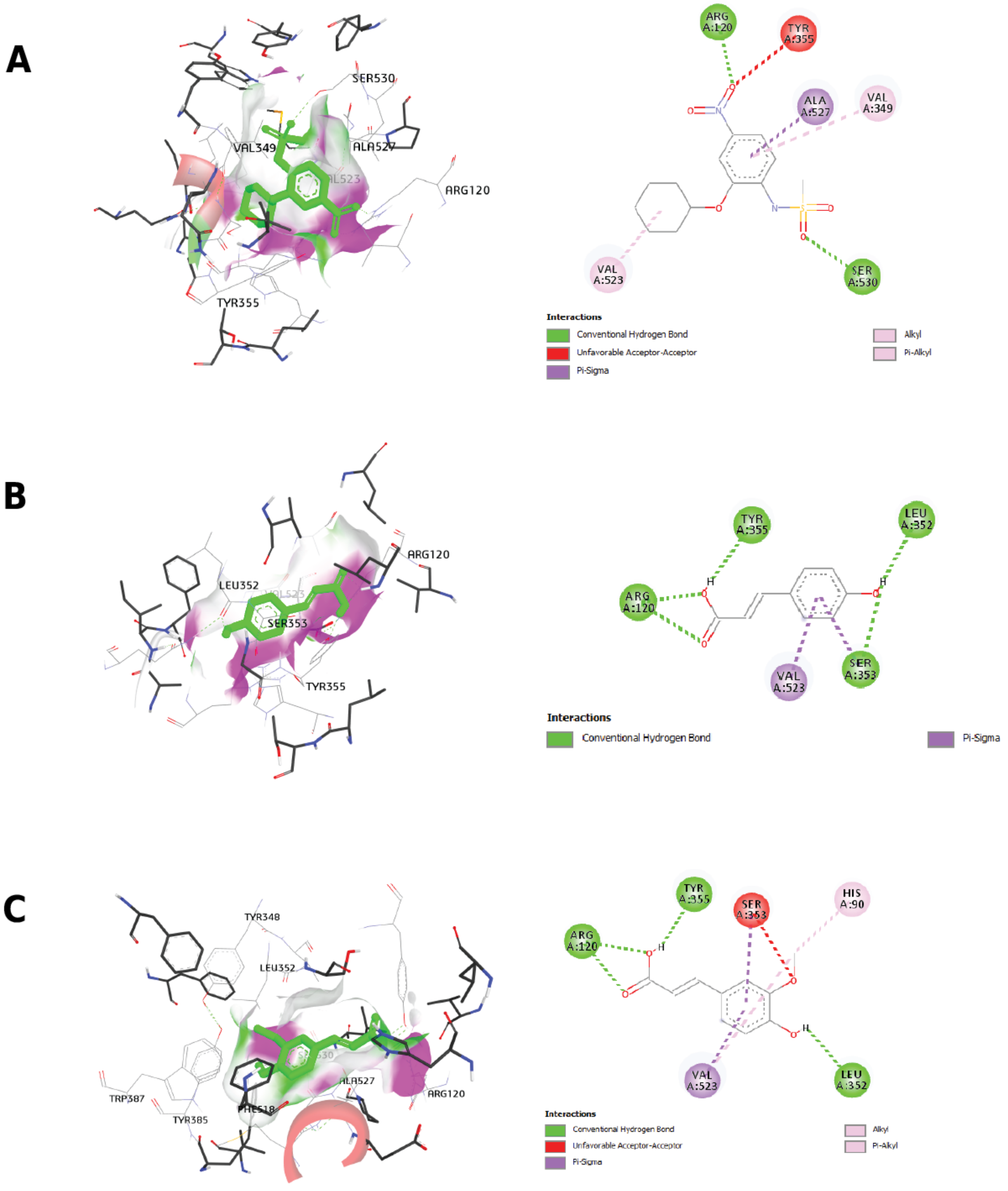

Figure S4. The established interactions of the most stable conformations of protein-ligand complex structures of: A – NS398, B – *p*-coumaric acid (*p*-CA), and C – ferulic acid (FA) and COX-2.

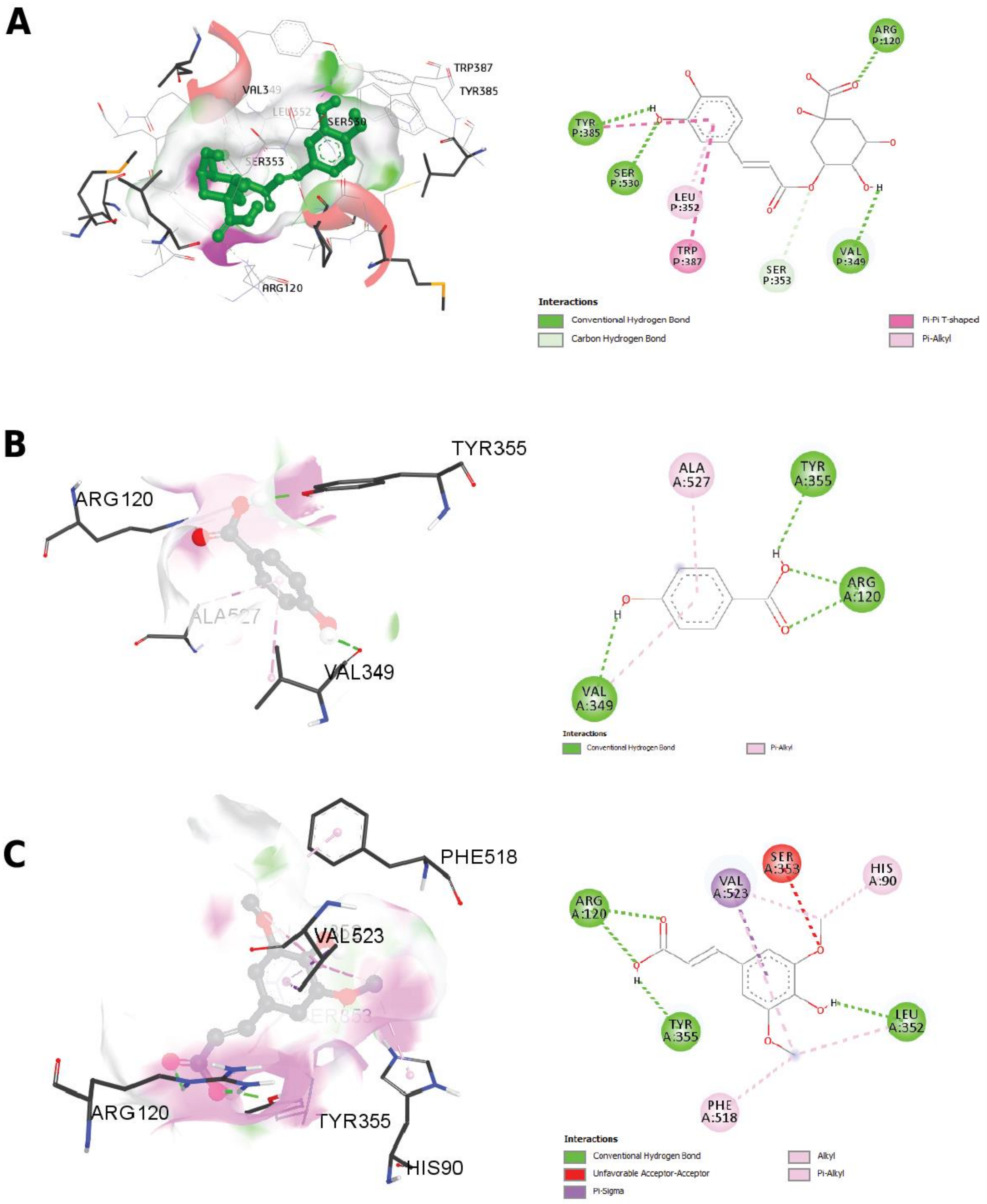

Figure S5. The established interactions of the most stable conformations of protein-ligand complex structures of: A – chlorogenic acid (CA), B – *p*-hydroxybenzoic acid (*p*-HBA), and C – synapic acid (SYN) and COX-2.

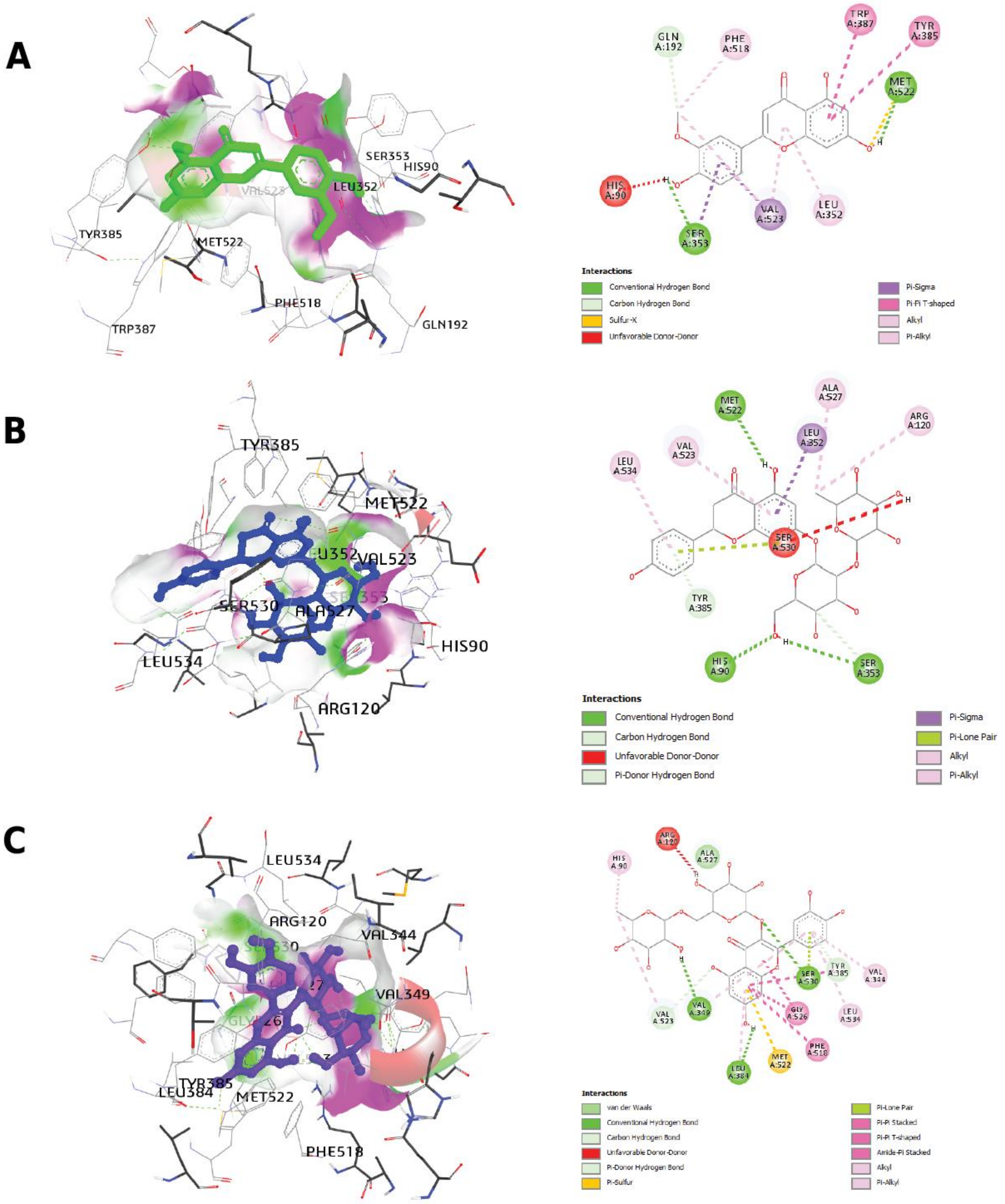

Figure S6. The established interactions of the most stable conformations of protein-ligand complex structures of: A – chrysoeriol (CHR), B – naringin (NAR), and C – rutin (RU) and COX-2.
